# HIV-1 Epidemic in the Caribbean Is Dominated by Subtype B

**DOI:** 10.1371/journal.pone.0004814

**Published:** 2009-03-12

**Authors:** Yuka Nadai, Lindsay M. Eyzaguirre, Anne Sill, Farley Cleghorn, Claudine Nolte, Manhattan Charurat, Santiago Collado-Chastel, Noreen Jack, Courtenay Bartholomew, Jean W. Pape, Peter Figueroa, William A. Blattner, Jean K. Carr

**Affiliations:** 1 Institute of Human Virology, School of Medicine, University of Maryland, Baltimore, Maryland, United States of America; 2 GHESKIO, Port-au-Prince, Haiti and Weill Medical College of Cornell University, New York, New York, United States of America; 3 Institute of Biomedical Studies, Santo Domingo, Dominican Republic; 4 Medical Research Foundation, Port of Spain, Trinidad and Tobago; 5 Ministry of Health, Kingston, Jamaica; University of Cape Town, South Africa

## Abstract

**Background:**

The molecular epidemiology of HIV-1 in the Caribbean has been described using partial genome sequencing; subtype B is the most common subtype in multiple countries. To expand our knowledge of this, nearly full genome amplification, sequencing and analysis was conducted.

**Methodology/Principal Findings:**

Virion RNA from sera collected in Haiti, Dominican Republic, Jamaica and Trinidad and Tobago were reverse transcribed, PCR amplified, sequenced and phylogenetically analyzed. Nearly full genomes were completed for 15 strains; partial *pol* was done for 67 strains. All but one of the 67 strains analyzed in *pol* were subtype B; the exception was a unique recombinant of subtypes B and C collected in the Dominican Republic. Of the nearly full genomes of 14 strains that were subtype B in *pol*, all were subtype B from one end of the genome to the other and not inter-subtype recombinants. Surprisingly, the Caribbean subtype B strains clustered significantly with each other and separate from subtype B from other parts of the pandemic.

**Conclusions:**

The more complete analysis of HIV-1 from 4 Caribbean countries confirms previous research using partial genome analysis that the predominant subtype in circulation was subtype B. The Caribbean strains are phylogenetically distinct from other subtype B strains although the biological meaning of this finding is unclear.

## Introduction

The Caribbean has the second highest HIV-1 prevalence in the world today, approximately 1% in adult men and women, exceeded only by sub-Saharan Africa [1]. There is quite a bit of variability between the different countries in the Caribbean, however. The national adult HIV prevalence rate exceeds 1% in Barbados, Dominican Republic, Jamaica and Suriname, 2% in the Bahamas, Guyana and Trinidad and Tobago, and is greater than 3% in Haiti.

The various epidemics in the Caribbean are driven predominantly by heterosexual intercourse, fuelled by commercial sex work in societies with widespread poverty. A background of stagnant tourism-dependent economies, early coitarche, high numbers of sexual partners for both sexes, high rates of sexually transmitted diseases, stigmatization and discrimination against sexual minorities are some of the many factors driving the Caribbean HIV-1 epidemic [2], [3], [4]. Homosexual intercourse is a minor factor in most of the epidemics, though the extent of its contribution is probably underestimated due to stigma. Injecting drug use drives the epidemics in only two locations: Bermuda and Puerto Rico [4].

Although international efforts to systematically collect, characterize, and classify HIV isolates from around the world have increased considerably, data on HIV-1 genetic variations in the Caribbean remains limited. Globally, nine subtypes and 34 circulating recombinant forms have been defined in HIV-1 Group M, which accounts for most of the HIV-1 pandemic. One of the earliest HIV-1 strains characterized by full-genome sequencing (RF) was from a Haitian woman living in the United States but since that time no full genomes have been sequenced to represent the epidemic(s) in the Caribbean [5]. Partial genome sequencing of viruses from multiple Caribbean islands have reported subtype B as virtually the only subtype present (with the exception of Cuba) [6]–[8]. Because subtype B is the most common subtype in the United States, its presence in the Caribbean would seem unremarkable, but in fact subtype B has never been the most common subtype in a heterosexual epidemic anywhere in the world [9]. As such, the epidemics in the Caribbean are unique.

The effort to more fully characterize the subtypes in circulation around the Caribbean was initiated by an examination of the viruses from HIV-positive patients from four countries: Dominican Republic (DO), Haiti (HT), Jamaica (JM) and Trinidad and Tobago (TT). The nearly full-length genomes of HIV-1 were amplified from the viral RNA and sequenced and represent the first nearly full-length sequences of HIV-1 from the Dominican Republic and Jamaica and the first in over a decade from Haiti and Trinidad and Tobago. To evaluate the genetic distribution pattern of HIV-1 in the Caribbean region, we analyzed phylogenetic relationships and genetic variability among HIV-1 strains isolated from patient samples collected between 2000 and 2005 in seroprevalence studies in Trinidad and Tobago, Jamaica, Haiti, and the Dominican Republic.

## Materials and Methods

### Study Subjects

Blood samples were drawn from patients attending the following centers: GHESKIO in Port-au-Prince, Haiti; Ministry of Health, Kingston, Jamaica; IDCP, Santo Domingo, Dominican Republic; Medical Research Foundation of Trinidad and Tobago, Port of Spain, Trinidad and Tobago. Patients were newly diagnosed as HIV(+) and had been infected for an unknown period of time. Serum was collected, frozen and sent to IHV, Baltimore, MD for analysis. All patients gave written informed consent and the study, including the notification of the subjects of their HIV status, was approved by the IRB of the University of Maryland, Baltimore, and the local participating institutions.

### Reverse Transcription and Amplification

The methods for RNA extraction, reverse transcription (RT), polymerase chain reaction (PCR) and the primers used for the nearly full-length PCR amplification have been previously described [10], [11]. Briefly, RNA was extracted from 140 µl of serum concentrated from 500 µl, using QIAamp Viral RNA Mini Assay (Qiagen, Valencia, CA). Reverse transcription of RNA was performed using SuperScript™ III RNase H^−^ Reverse Transcriptase (Invitrogen, Carlsbad, CA) with RT3474R and UNINEF7′ primers for the partial *pol* region and the nearly full-length genome amplification, respectively. cDNA synthesized with UNINEF7′ as primer was used as template for the nearly full-length PCR amplification with limiting-dilution methods [11]. This method utilizes one, two or three overlapping amplicons to cover most of the HIV genome, from 795 to 9180 on HXB-2 (Genbank Accession No. K03455) and is routinely successful with well preserved serum having a viral load of 10^5^ or greater. The amplicon does not, however, include the U5, R or U3 components of the viral RNA.

Specifically, reverse transcription of the RNA was performed by priming with UNINEF7′ (5′-GCACTCAAGGCAAGCTTTATTGAGGCTT-3′) close to the 3′ end of the viral RNA or by VIF-VPUoutR1 (5′-GGTACCCCATAATAGACTGTRACCCACAA-3′) in *vpu* [11]. The extracted RNA (3 µl) was reverse transcribed in a total volume of 20 µl with 500 mM dNTP, 2.5 mM primer, 1× RT buffer, 5 mM MgCl2, 10 mM DTT, 40 U RnaseOUT, and 400 U SuperScript™ III RNase H^−^ RT (Invitrogen, Carlsbad, CA). The RNA, primer and dNTPs were first incubated at 65°C for 5 minutes, then the remaining reagents were added for cDNA synthesis at 50°C for 2 hours, followed by 85°C for 5 minutes. Then 2 U E. coli RNase H (Invitrogen, Carlsbad, CA) was added, and the reaction tubes were incubated at 37°C for 20 minutes followed by 70°C for 15 minutes.

Two or three regions of the viral genome were independently amplified from the cDNA to contain a nearly full-length genome of HIV-1. The two-amplicon strategy consisted of one amplicon of about 2.6-kb consisting of *gag* and part of *pol* (nts 769-3338 HXB-2 (Genbank Acc No: K03455)) and another amplicon of approximately 7.0-kb stretching from the 5′ end of *pol* to the middle of *nef* (nts 2143–9181 HXB-2). A three-amplicon strategy was used as needed. The first amplicon was the *gag*-*pol* amplicon described above, the second spanned *pol* to *vpu* (nts 2483–6231 HXB-2) and the third *env* to *nef* (nts 5861–9181 HXB-2). The reaction volume was 50 µl, containing 1× PCR buffer, 350 µM dNTP mixture, 0.4 µM of each primer and 5 U Expand Long Template PCR enzyme mixture (Roche Diagnostics, Indianapolis, IN). Cycling conditions for the first round were: 94°C for 2 minutes and then 10 cycles of (94°C 10 s, 60°C 30 s, 68°C 3 min), then 20 cycles of (94°C 10 s, 55°C 30 s, 68°C 3 min) followed by 68°C for 10 minutes. For the second round, cycling conditions were the same, except that the annealing temperature for the first 10 cycles was 65°C and the incubation at 68°C was for 8 minutes.

The products of reverse transcription with the primer RT3474R were employed for amplification of the partial *pol* region. The first-round amplification was done with 2 primers: Pro5F (5′-AGAAATTGCAGGGCCCCTAGGAA) and RT3474R (5′-GAATCTCTCTGTTTTCTGCCAG), using AmpliTaq Gold (Applied Biosystems, Foster City, CA) and 2 mM of MgCl_2_ in a total volume of 50 µL. The second-round amplification was completed using the following 2 primers: Pro3F (5′-AGANCAGAGCCAACAGCCCCACCA) and ProRT (5′- TTTCCCCACTAACTTCTGTATGTCATTGACA). The first-round amplification reactions began with a “hot start” at 95°C for 10 minutes to activate the polymerase, followed by 30 cycles for 30 seconds at 94°C, 30 seconds at 55°C, and 1.5 minutes at 72°C, then a step at 7 minutes at 72°C was followed to end the cycles. For the second-round amplification, the cycling conditions were the same except for an annealing temperature of 58°C and the use of 40 cycles instead of 30. The partial *pol* amplicon contained the coding sequences for protease and part of reverse transcriptase (RT), corresponding to nts 2167 through 3307 on HXB-2.

Amplified DNA for the partial *pol* and nearly full-length regions were purified and sequenced in the Applied Biosystems 3130xl automated sequencer using Big Dye terminators (Applied Biosystems), and the sequences were assembled with Sequencher v4.6 (Gene Codes Corporation, Ann Arbor, MI). When multiple amplicons were used, they were not merged unless the overlapping sequences were within 2% of each other. Nearly full genome and partial *pol* sequences were deposited in Genbank under accession numbers EU839596–EU839610 and EU439709–EU439774, respectively.

#### Analysis

A multiple alignment of the newly derived protease/RT sequences and nearly full-length genome sequences with selected reference sequences was constructed, consisting of 1069 nts and 8364 nts, respectively. Phylogenetic trees were generated, and the consistency of branching order was evaluated using SEQBOOT, DNADIST, NEIGHBOR, and CONSENSE modules of the Phylogeny Inference Package (V3.52c). Recombinant analysis was performed with Simplot, version 3.4, and alignment examination was used to determine precise breakpoints [12]. After breakpoint identification, each segment was extracted and analyzed phylogenetically to confirm the assignment of subtype; breakpoint locations were designed relative to HXB-2. The Kimura 2-parameter method was used to calculate pairwise genetic distances. Analysis of the protease-RT sequences for mutations that might lead to resistance to antiretroviral drugs was performed using the Stanford University Database (http://hivdb.stanford.edu/hiv).

The nine genes of HIV-1 were translated from the nucleotide sequence and visually examined to identify sites that might distinguish the Caribbean strains. The prevalence of particular amino acids were compared between the Caribbean sequences and reference B sequences using Fisher's Exact Test without correcting for multiple comparisons.

## Results

**Figure 1:**
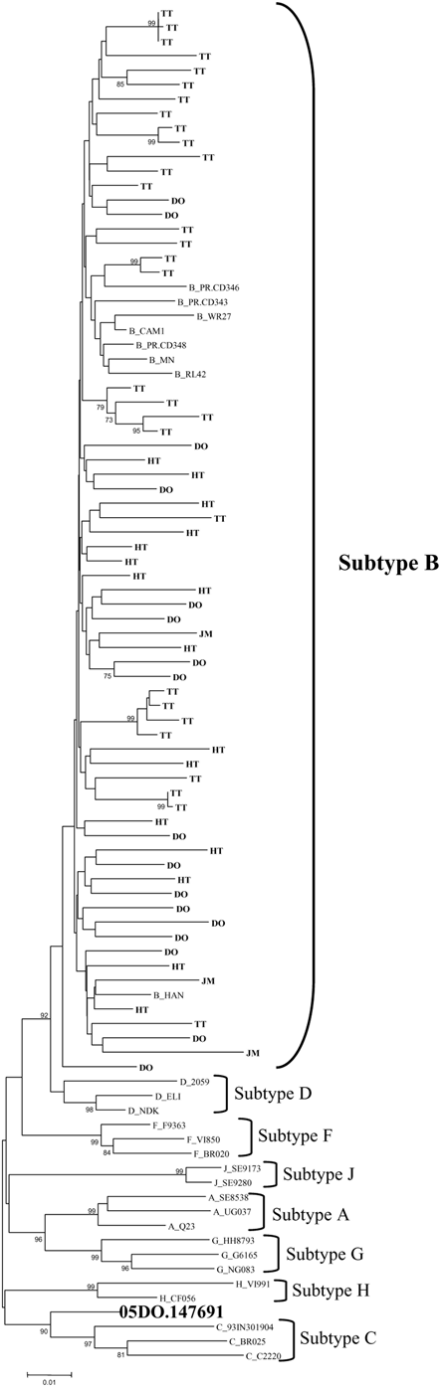
Phylogenetic analysis of 67 partial *pol* sequences of HIV-1 from Caribbean countries: Trinidad and Tobago (TT), Dominican Republic (DO), Haiti (HT) and Jamaica (JM). A neighbor-joining phylogenetic tree was built using the Kimura 2-parameter model and significant parsimony bootstrap values (>70%) were placed next to the nodes. The genetic distance corresponding to the lengths of the branches is shown by the scale below the tree. Reference samples are those named, preceded by the subtype; one of the study samples (the B/C recombinant) is also named.

A total of 67 partial *pol* sequences of HIV-1 from the four Caribbean countries were analyzed: Trinidad and Tobago (n = 30), Dominican Republic (n = 18), Haiti (n = 16) and Jamaica (n = 3). Phylogenetic analysis classified 66 partial *pol* sequences as HIV-1 subtype B and one, from the Dominican Republic, as a recombinant between subtypes B and C (Figure 1). Based on the genetic analysis, some of the Trinidad samples were probably epidemiologically linked but aside from those clusters there was no clustering within Caribbean subtype B by country, island or language.

Of the 67 samples with partial *pol* sequences, 15 specimens were also successfully amplified for the nearly full-length genome from serum RNA. The 15 that underwent full-length analysis consisted of the one non-B and those that produced full length ampicons readily. Previous experience with the primers suggests that failure to amplify was not due to primer mismatch, but rather to sample quality. Fourteen strains were non-recombinant subtype B; all strains were analyzed for evidence of intersubtype recombination and none was observed. The nearly full-length sequence of the B/C recombinant strain revealed that it was a unique recombinant form which was subtype B in protease and the amino terminus of RT, while the rest of the genome was subtype C. The sequence did not cluster with CRF31_BC or any other B/C recombinant in genbank.

**Figure 2:**
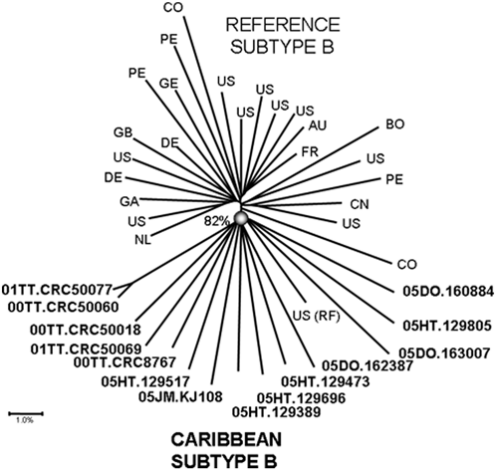
Phylogenetic analysis of 14 nearly full genome sequences of HIV-1 Subtype B from Caribbean countries: Trinidad and Tobago (TT), Dominican Republic (DO), Haiti (HT) and Jamaica (JM). Reference subtype B sequences are indicated by the country of origin; they are: US: MN, JRCSF, SF2, WR27, P896, NY5, BCSG3C, YU2, RF, U23487; DE: D31, HAN; GB: CAM1, study sequences (bold) are identified by name. A neighbor-joining phylogenetic tree was built using the Kimura 2-parameter model and significant parsimony bootstrap values (>70%) were placed next to the nodes. The genetic distance corresponding to the lengths of the branches is shown by the scale below the tree.

When analyzed with other subtype B strains from the pandemic, the 14 nearly full-length sequences of the subtype B strains from the Caribbean clustered together significantly with a bootstrap value of 82% (Figure 2). The only ‘non-Caribbean’ subtype B was RF, a virus collected from an Haitian woman in the United States [5]. Aside from one cluster between two of the Trinidad samples there was no significant clustering within the Caribbean strains, either by country, island or language. Analysis of the nine genes of HIV-1 for mutations more common in the Caribbean strains than in subtype B reference strains revealed a handful of sites that significantly distinguished the two populations (Table 1).

**Table 1:** Amino acid sites that differ between the Caribbean B strains and the subtype B reference strains.

Gene	Location	Mutation	Caribbean B	Reference B	p
gag			(n = 25)	(n = 19)	
	matrix	D102E	23	11	0.01
	capsid	I159V	9	15	0.005
pol			(n = 14)		
	signal peptide	N21TDA	8	18	0.03
	protease	K97R	1	13	0.0004
rev			(n = 14)		
		V102I	2	13	0.002
env			(n = 14)		
	V3 loop	T319-	5	0	0.008
	C5	N474D	5	15	0.01
	C5	K476RM	5	17	0.002

Analysis of the protease/RT sequences revealed a lack of major drug resistance-conferring mutations among the Caribbean HIV-1-infected samples except for one sample from the Dominican Republic. Tropism for co-receptor usage of HIV-1 was determined *in silico* for 22 partial *env* sequences from the study population. The PSSM procedure was used to predict the phenotype [13]. Only 2 out of the 22 sequences had a preference for CXCR4 rather than CCR5, both of them from the Dominican Republic.

We analyzed the V3 loop of the samples and there was a deletion of threonine in the V3 loop in 40% of the Caribbean samples overall, and this was more common in samples from Trinidad and Tobago (67%) than in the other countries, where it ranged from 20% (JM), to 33% (DO). Overall, this mutation is associated preferentially with the Caribbean strains (env T319-, Table 1).

## Discussion

Nearly full-length genome sequencing has been tremendously helpful in understanding the HIV epidemics in many parts of the world; when inter-subtype recombinants are circulating, partial genome sequencing can be very misleading. To date, the only HIV viral genomes collected in the Caribbean that have been fully sequenced were from Cuba [14]. Partial genome sequencing by many has shown that outside of Cuba the epidemic was dominated by subtype B, the strain of HIV-1 common in the developed world [2], [6], [8], [15], [16]. It was to improve our knowledge about the strains in the Caribbean that nearly full genome amplification and sequencing was performed for samples from 4 countries: Trinidad and Tobago, Jamaica, Dominican Republic and Haiti.

Based on the sequence of the protease and RT region of *pol*, the majority of strains (66/67, 98.5%) were subtype B, although one, from the Dominican Republic, was a B/C recombinant. Phylogenetic analysis of this region revealed no clustering of Caribbean sequences distinct from non-Caribbean, or between one country and another.

Analysis of the nearly full genome sequences of the 14 subtype B strains demonstrated no evidence of inter-subtype recombination. Of note, however, is the fact that the region of the genome that was sequenced did not include the parts of the RNA that form the LTR in the provirus, consequently it is still possible that the LTR could be from a non-B subtype, although not likely. Full genome analysis of the subtype B strains did support the distinct clustering of the subtype B Caribbean sequences separate from those from the pandemic, including South America. This is surprising because there is undoubtedly a great deal of human traffic between the U.S. and Europe, on the one hand, and the Caribbean, on the other. In an attempt to locate the part of the genome that might be contributing to the monophyletic clustering, a sliding window examination of 8.5 kb revealed no specific location on the genome that was responsible. There were, however, isolated sites that distinguished the Caribbean strains from others. Shown in Table 1, there were some amino acid mutations that significantly distinguished the two populations. In comparisons such as this, it is appropriate to consider the fact that these comparisons are being done for each of the >3000 amino acids in the HIV-1 genome, and thus the statistical significance should be adjusted. If that adjustment were done, no amino acid mutations would statistically distinguish the two populations. As an exploratory exercise, however, it is valuable to explore where the phylogenetic signal may be coming from. The first site, gag D102E, occurs in the matrix protein and is associated with Elite Controllers [17]. Another Caribbean-specific mutation has already been reported: the presence of a threonine deletion in the V3 loop of Trinidad samples (env T319-) [8]. Most of the other changes were conservative, and are not known to be associated with specific phenotypic differences. These sites and others were so uncommon that phylogenetic clustering only achieved statistical significance when the full genome was analyzed (data not shown). It is unclear what the biological importance of this phylogenetic distinction is but this question could be explored *in vitro*.

There are two possible explanations for the phylogenetic clustering of the Caribbean strains. The first is that it can be the result of founder effects from when the epidemic first began that are still detectable 20 years later. There are several arguments against that theory: given the human traffic in and out of the Caribbean, it is extremely unlikely that a founder effect would continue so long. Another fact that argues against the founder effect is that there is no clustering of samples by country or language, as you would expect if there were different founder viruses in different islands. Another explanation is that there may be some form of consistant selection pressure on these viruses that is not as strong in subtype B viruses from the developed world. As mentioned earlier, the Caribbean is the only region of the world that has a robust heterosexually-driven epidemic of subtype B. In Thailand, South Africa and Argentina, concentrated epidemics in high risk populations preceded generalized epidemics in the general public. In each of these cases, subtype B was the subtype common in the concentrated epidemic and a non-B subtype replaced it as the epidemic grew [9], [16]. Subtype B viruses from the Caribbean may have acquired features that have enabled it to generate a more ‘successful’ heterosexual epidemic.

The data presented here have limitations: the samples used were not collected using any systematic sampling method and might not adequately represent the strains in these four countries. The number of full length genome sequences that could be performed was limited, and the selection of those was determined by ease of amplification, which could bring in a consistant bias against certain strains. These primers have been used successfully for many subtypes and the likelihood of sequence bias is not high, however. In general, the study was limited by the small sample size, and this would have been more consequential if the molecular epidemiology had been more complex. The findings, however, are consistant with the findings of others based on partial genome sequences and this lends support to their validity.

The HIV epidemic in the Caribbean is second only to sub-Saharan Africa in the prevalence of infection in the generalized population [1]. The complexities of the region, with its different islands, languages and cultures, has presented a challenge for coordinating research activities throughout the region, difficulties that are compounded by the low standard of living for many of the countries. As anti-retroviral drugs are deployed across the region, it becomes even more important to have a solid baseline understanding of the molecular epidemiology of HIV in the various countries, and to begin to track the emergence of drug resistance.
